# Novel Compound Heterozygous Mutations in *MYO7A* Associated with Usher Syndrome 1 in a Chinese Family

**DOI:** 10.1371/journal.pone.0103415

**Published:** 2014-07-31

**Authors:** Xue Gao, Guo-Jian Wang, Yong-Yi Yuan, Feng Xin, Ming-Yu Han, Jing-Qiao Lu, Hui Zhao, Fei Yu, Jin-Cao Xu, Mei-Guang Zhang, Jiang Dong, Xi Lin, Pu Dai

**Affiliations:** 1 Department of Otolaryngology, Head and Neck Surgery, PLA General Hospital, Beijing, P. R. China; 2 Department of Otolaryngology, Hainan Branch of PLA General Hospital, Sanya, P. R. China; 3 Department of Otolaryngology, Emory University School of Medicine, Atlanta, Georgia, United States of America; 4 Department of Otolaryngology, the Second Artillery General Hospital, Beijing, P. R. China; 5 Xi’an Research Institute of Hi_tech, Hongqing, Xi’an, Shaanxi, P. R. China; National Eye Institute, United States of America

## Abstract

Usher syndrome is an autosomal recessive disease characterized by sensorineural hearing loss, age-dependent retinitis pigmentosa (RP), and occasionally vestibular dysfunction. The most severe form is Usher syndrome type 1 (USH1). Mutations in the *MYO7A* gene are responsible for USH1 and account for 29–55% of USH1 cases. Here, we characterized a Chinese family (no. 7162) with USH1. Combining the targeted capture of 131 known deafness genes, next-generation sequencing, and bioinformatic analysis, we identified two deleterious compound heterozygous mutations in the *MYO7A* gene: a reported missense mutation c.73G>A (p.G25R) and a novel nonsense mutation c.462C>A (p.C154X). The two compound variants are absent in 219 ethnicity-matched controls, co-segregates with the USH clinical phenotypes, including hearing loss, vestibular dysfunction, and age-dependent penetrance of progressive RP, in family 7162. Therefore, we concluded that the USH1 in this family was caused by compound heterozygous mutations in *MYO7A*.

## Introduction

Usher syndrome is a clinically and genetically heterogeneous recessive disease with a worldwide prevalence of 1 in 16,000–50,000 [1]. Based on the severity and progression of hearing loss, age at onset of retinitis pigmentosa (RP), and presence or absence of vestibular impairment, the majority of Usher syndrome cases can be classified into three clinical subtypes. The most severe is Usher syndrome type 1 (USH1), which is characterized by severe-to-profound congenital hearing loss, absence of vestibular function in most cases, and prepubertal-onset retinal degeneration, with impaired night vision and gradual restriction of the visual fields diagnosed as RP. In most populations, USH1 accounts for approximately one-third of Usher syndrome patients. Usher syndrome type II (USH2) has moderate-to-severe sensorineural hearing loss that is stable in most cases, normal vestibular function, and RP, whereas patients with type III (USH3) have moderate sensorineural hearing loss with progression to acquired deafness, progressive vestibular dysfunction, and RP. Cohen et al has reported that among the deaf population, the proportion of patients with USH may be as high as 10%, making Usher syndrome an important diagnosis in clinical practices [2]. Molecular genetic testing can confirm or exclude Usher syndrome at an early age, even before the onset of visual problems [3].

Seven loci have been mapped for USH1 (*USH1B-USH1H*). Five causative genes have been identified: *USH1B*, encoding myosin VIIa; *USH1C*, encoding harmonin (USH1C); *USH1D*, encoding cadherin 23 (CDH23); *USH1F*, encoding protocadherin 15 (PCDH15); and *USH1G*, encoding SANS. Mutations in these genes affect the pressure-sensitive stereocilia of the inner ear (Hereditary Hearing Loss Homepage, http://hereditaryhearingloss.org/) and do not occur at the same frequencies across ethnicities. Of all pathogenic mutations, 29∼55% are in the *MYO7A* gene [4], [5], [6], [7], [8], [9], [10], [11].

Collectively, the five USH1 genes comprise 183 coding exons (http://www.genome.ucsc.edu/). A comprehensive molecular diagnosis of Usher syndrome has been hampered both by the genetic heterogeneity of the disease and the large number of exons of known Usher syndrome genes. Next-generation sequencing (NGS) is a revolutionary technology that allows the simultaneous screening of mutations in a large number of genes. It is cost effective compared to classical strategies of linkage analysis and direct sequencing when the number or size of genes is large [12]. Therefore, targeted deafness gene capture combined with NGS provides opportunities to identify causative mutations and new Usher syndrome genes using a limited number of patient samples [13], [14], [15], [16], [17], [18].

The *MYO7A* gene has 49 exons, spans approximately 87 kb of genomic sequence on chromosome 11q13.5, and encodes the actin-based motor protein myosin VIIa. The protein consists of 2215 amino acids and contains an N-terminal motor domain, a neck region containing several IQ motifs, a short predicted coiled coil domain, a MyTH4 domain, a FERM domain, an SH3 domain, and a second C-terminal MyTH4-FERM tandem domain [19]. In humans, myosin VIIa is expressed in a variety of cells, including the inner ear hair cells, retinal pigment epithelium, and photoreceptor cells of the retina [20]. Different roles have been postulated for myosin VIIa in the inner ear, such as participation in mechano-transduction in hair cells and differentiation and organization of hair cell stereocilia [21]. In the human retina, myosin VIIa functions actively in the migration of retinal pigment epithelium, photoreceptor cells, and opsin transport [22], [23]. Mutations in this gene have been reported to cause Usher syndrome type 1B (USH1B) and non-syndromic deafness (DFNB2, DFNA11) [24], [25], [26].

In this study, we performed large-scale mutation screening of 131 known deafness-related genes, including 5 USH1 genes, in a Chinese family (no. 7162) diagnosed with USH1 and identified two compound heterozygous disease-segregating mutations in the *MYO7A* gene: a known missense mutation c.73G>A (p.G25R) and a novel nonsense mutation c.462C>A (p.C154X).

## Materials and Methods

### Clinical data

Family 7162 is a Chinese family clinically diagnosed with autosomal recessive USH1. To identify candidate mutations, DNA samples were obtained from eight members of family 7162 and 219 ethnicity-matched controls. Written informed consent was obtained from each subject or their guardians. The study protocol, including the consent procedure, was performed with the approval of the Ethics Committee of Chinese PLA General Hospital. A medical history was obtained using a questionnaire regarding the following aspects: age at onset, evolution, symmetry of the hearing impairment, presence of tinnitus, medication, noise exposure, possible head or brain injury, use of aminoglycoside antibiotics, and other relevant clinical manifestations. A physical examination, otoscopy, and pure tone audiometric examination (at frequencies from 250 to 8000 Hz) were performed to identify the phenotype. Immittance testing was used to evaluate the middle-ear pressure, ear canal volume, and tympanic membrane mobility. Unaffected phenotype status was defined by a threshold lower than the age- and gender-matched 50^th^ percentile values for all frequencies measured. The physical examinations of all members revealed no signs of systemic illness or dysmorphic features. Computed tomography (CT) of the temporal bone was performed in the index patient. A diagnosis of profound sensorineural hearing impairment was made according to the ICD-10 criteria based on the audiometric examination.

Vestibular functions were evaluated using the tandem gait and Romberg tests. The ocular examination included the best-corrected visual acuity, slit lamp examination, and detailed stereoscopic fundoscopy. The electroretinogram (ERG) was measured according to the standards of the International Society for Clinical Electrophysiology of Vision [27] beginning after 30 min of dark adaption using 10-µs xenon flashes in a Ganzfeld bowl. The pupils were dilated fully using 10% phenylephrine HCl and 1% tropicamide, and Burian-Allen bipolar corneal electrodes were applied after topical anesthesia with 5% proparacaine HCl.

### Deafness gene capture and Illumina library preparation

Genomic DNA (gDNA) was extracted from peripheral blood using a blood DNA extraction kit, according to the manufacturer’s protocol (Tiangen, Beijing, China). Three prevalent deafness-associated genes, *GJB2*, *SLC26A4*, and mtDNA*12SrRNA,* were first screened for mutations in all participating family members. Then, we sequenced all of the coding exons plus ∼100 bp of the flanking intronic sequences for 131 deafness genes and ∼5 kilobases of *GJB2* regulatory sequences (Table S1) in three affected members (II:1, II:2, II:4) and three unaffected members (II:3, I:1, I:2) of family 7162.

gDNA quality was evaluated using the optical density ratio (260/280 ratio) and gel electrophoresis imaging. High-molecular-weight gDNA (∼3 µg) was fragmented ultrasonically using an E210 DNA-shearing instrument (Covaris; Woburn, MA, USA) to an average size of 300 base pairs (bps). The Covaris protocol was set at 3-min total duration, duty cycle 10%, intensity 5, and 200 cycles per burst.

Fragmented gDNA libraries for Illumina GAII sequencing were prepared with the NEBNext™ DNA Sample Prep Master Mix set (E6040, New England BioLabs; Ipswich, MA). End repair of DNA fragments, the addition of a 3′ adenine (A), adaptor ligation, and reaction cleanup were performed following the manufacturer’s protocol. The libraries were cleaned and size-selected using the AMPure DNA Purification kit (Beckman Agencourt; Danvers, MA, USA). The ligated product (20 ng) was amplified for 14 PCR cycles with the Illumina PCR primers InPE1.0, InPE2.0, and indexing primers, following the manufacturer’s instructions.

For targeted enrichment of deafness genes, the Illumina library DNA was purified with a QIAquickMinElute column and eluted into 50 µL of hybridization buffer (HB, Roche NimbleGen; Madison, WI, USA). The barcoded Illumina gDNA libraries (0.5 µg) were incubated in with probes to enrich for the targets in solution. More details of capture probe validation and preparation can be found in our previous study [28]. Nonspecific DNA fragments were removed after six washing steps in a washing buffer (Roche NimbleGen, Madison, WI). The DNA bound to the probes was eluted by incubating it with NaOH (425 mL, 125 mM) for 10 min. The eluted solution was transferred to a 1.5-mL Eppendorf tube containing 500 µL of neutralization buffer (QIAGEN PBI buffer). The neutralized DNA was desalted and concentrated on a QIAquick MinElute column and eluted into 30 µL in EB buffer. To increase the yield, we typically amplified 5 µL of eluted solution for 12 PCR cycles using the Illumina PCR primers InpE1.0 and 2.0. The enrichment of the targeted deafness gene sequences was assessed using quantitative PCR (qPCR) by comparing the growth curves of captured and non-captured samples [29]. Barcoded libraries of captured samples were pooled and paired-end Illumina sequencing was performed using the Illumina HiSeq system (Illumina; San Diego, CA, USA). Details of the bioinformatics analysis methods have been published [29]. Sequence read data of the affected subjects in family 7162 has been deposited into Sequence Read Archive (http://www.ncbi.nlm.nih.gov/sra webcite; accession number SRR1296682).

### Mutational analysis of MYO7A

The segregation of the *MYO7A* c.73G>A and c.462C>A mutations was tested in eight family members (I:1, I:2, II:1, II:2, II:3, II:4, III:1 and III:3), including the five whose gDNAs have been subjected to 131 deafness-associated gene NGS analysis, using PCR (primer sequences available on request) followed by bidirectional Sanger sequencing of the amplified fragments (ABI 3100; Applied Biosystems). Nucleotide alterations were identified by sequence alignment with the *MYO7A* GenBank sequence using the GeneTools software. In addition, sequences from 219 ethnicity-matched negative samples were examined.

Multiple sequence alignment was performed using ClustalW2 with the default settings and the sequences *NP_000251.3* (Homo sapiens), *XP_001087868.2* (Macacamulatta), *XP_003313297.2* (Pan troglodytes), *XP_002693553.2* (Bostaurus), *XP_542292.3* (Canis lupus), *NP_001243010.1* (Musmusculus), *NP_703203.1* (Rattusnorvegicus), *XP_417277.3* (Gallusgallus), and *NP_694515.1* (Daniorerio).

### Model building and structural-based analysis

Three-dimensional (3D) modeling of the human wild-type and p.G25R mutation was carried out using SWISS-MODEL, an automated homology modeling program (http://swissmodel.expasy.org/workspace/). This study used the automatic modeling approach to model the complete human myosin VIIa protein, including its 2215 amino acids (NP_000251.3) with or without the mutations. Data obtained from the homology models were visualized using Swiss-PdbViewer 4.1.

## Results

### Family and clinical evaluations

**Figure 1:**
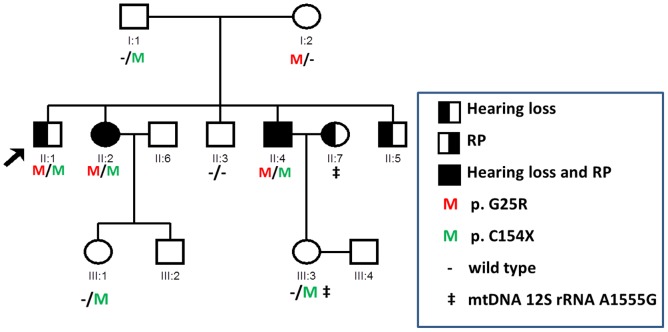
Pedigree of Chinese Family 7162 with Recessive USH1 and segregation of the mutations in *MYO7A*. The proband is indicated by an arrow. Subject I:1, I:2, II:1, II:2, II:3 and II:4 were tested by NGS. gDNA from II:5 is not available.

**Figure 2:**
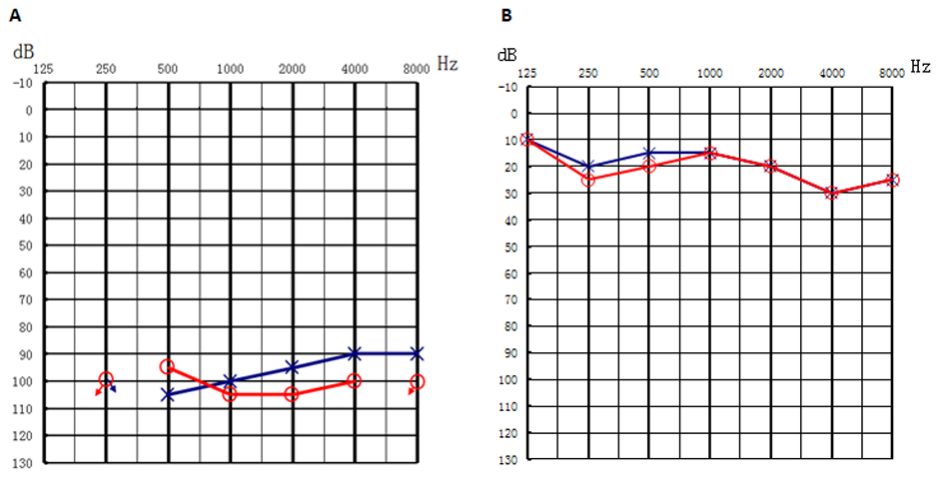
Audiogram showed bilateral profound sensorineural hearing loss of affected subjects II:4 and normal hearing of subject II:3 (red, right ear; blue, left ear).

We analyzed a Chinese USH1 family (no. 7162, Figure 1), which includes four affected siblings: II:1 (male, 62 years old), II:2 (female, 60 years old), II:4 (male, 54 years old) and II:5 (male, 52 years old), one unaffected siblings II:3 (male, 57 years old), two unaffected parents (I:1, 83 years old and I:2, 80 years old), and two unaffected daughters (III:1, 30 years old and III:3, 27 years old). For each subject, the diagnosis was established from the medical history and a detailed evaluation of vision, vestibular function, and hearing. Hearing loss is congenital and stable. Audiograms of the affected siblings showed that the hearing loss was bilateral and profound (Figure 2). Immittance testing demonstrated normal and bone conduction values equal to the air conduction measurements, suggesting sensorineural hearing impairment. High-resolution CT of the temporal bone and the brain in the affected subject II:4 was normal, excluding inner-ear gross malformations. The physical examinations of all participating members revealed no signs of systemic illness or dysmorphic features. Affected individuals did not have obvious delayed gross motor development. This phenotype is consistent with that reported for USH1. For the affected subject II:5, gDNA and examinations were unavailable. The penetrance of hearing loss and RP was 100% and 50%, respectively (Figure 1).

**Figure 3:**
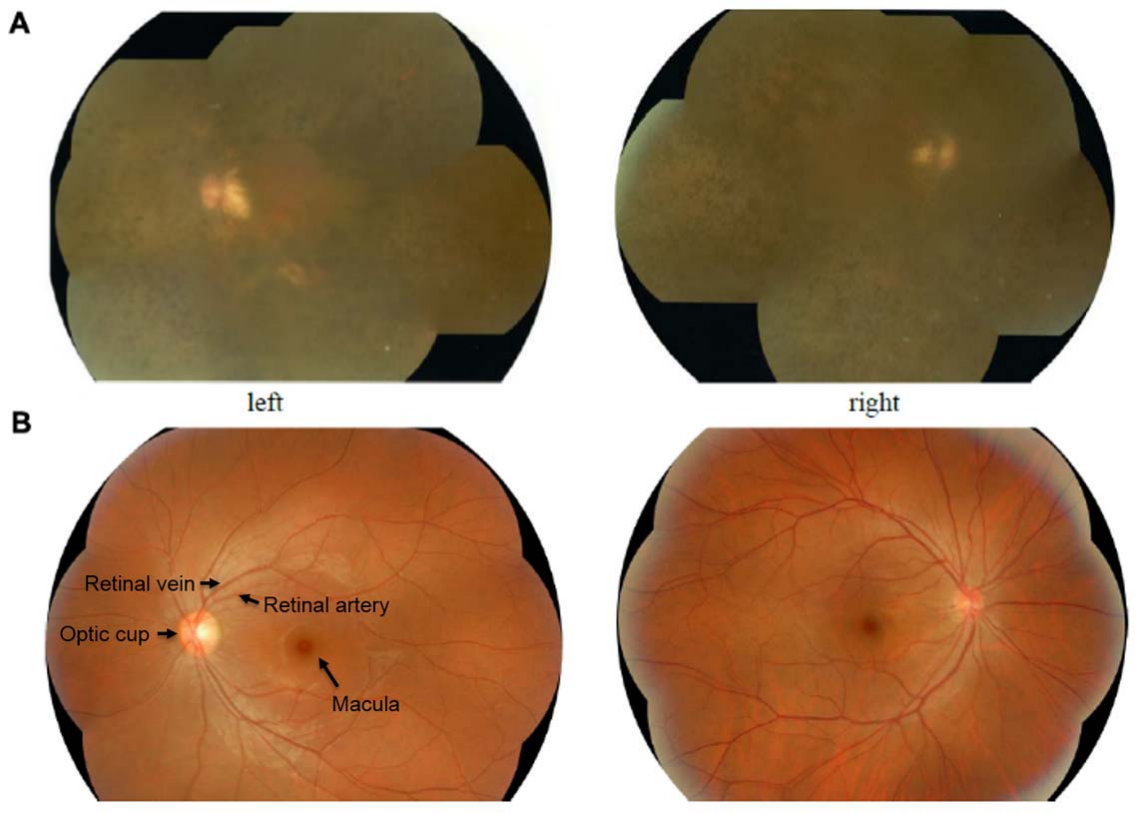
Composite image of the fundus photographs of individuals from family 7162. A: The appearance of the fundus in one patient (II:4) with RP at 54 year old shows typical retinal degeneration with obvious waxy pallor of the optic discs, attenuation of the retinal vessels, irregular pigment clumps in the retina. B: Fundus photographs of patient II:1 without RP at 62 years old shows bright optic disc where blood vessels converge.

For affected subject II:4, tandem walking was abnormal and the Romberg test was positive. Difficulty with night vision was observed at a very young age, and constriction of the visual field was apparent in the second decade of life, which likely occurred earlier. The ophthalmoscopic examination demonstrated obvious waxy pallor of the optic discs, attenuation of the retinal vessels, and bone spicule-type pigment deposits (Figure 3). The ERG wave amplitudes of patient II:4 were undetectable from the baseline.

### Targeted deafness gene capture and massively paralleled sequencing

One subject, II:7, is married to one of the sibling, II:4, and was identified to carry a homogeneous mtDNA*12S rRNA*A1555G mutation (Figure 1). This mutation was passed to daughter III:3. Subject II:7 had a history of aminoglycoside administration and is hearing impaired. All subjects were screened first for mutations of *GJB2* and *SLC26A4*, which have a predominant presence in genetic hearing loss. Both genes were excluded. We subsequently sequenced all of the coding exons plus ∼100 bp of the flanking intronic sequence of 131 deafness genes in three affected (II:1, II:2, II:4) and two unaffected members of family 7162 (I:1, II:3). Four variants leading to amino acid change were detected in the *MYO7A*:c.73G>A (G25R), c.462C>A (C154X), c.47T>C (L16S), and c.4996A>T (S1666C). Of these, two variants c.47T>C (L16S) and c.4996A>T (S1666C) were found in the homozygous state in unaffected members as well as in the affected members, suggesting that these two variants are non-pathogenic. In contrast, the *MYO7A* compound heterozygous variants c.73G>A (p.G25R) and c.462C>A (p.C154X) co-segregated in all affected family members tested.

### Mutation analysis

**Figure 4:**
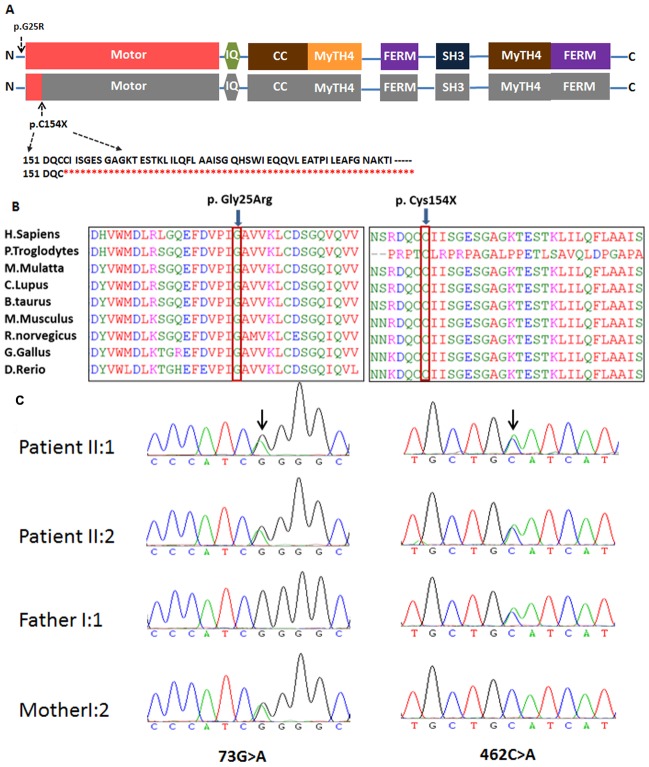
Domain structure, conservation analysis and mutational analysis of *MYO7A* in family 7162. A: Domain structure of myosin VIIa showing the nonsense mutation introduces a premature stop codon which is predicted to truncate the protein within the N-terminal motor domain. B: Protein alignment showing conservation of residues myosin VIIaG25 and C154 across nine species. Two mutations both occurred at evolutionarily conserved amino acids (in red box). C: DNA sequencing profile showing the c.73G>A and c.462C>A mutations in *MYO7A*. Both variants co-segregated with the clinical phenotype and c.462C>A were absent in 219 ethnicity-matched controls.

Using Sanger sequencing, eight participating members (three affected, five unaffected) in family 7162 were genotyped to identify the mutations. Compound heterozygous c.73G>A (p.G25R) and c.462C>A (p.C154X) variants of *MYO7A* were found in three affected family members (II:1, II:2, and II:4) (Figure 1 and 4C); these were considered pathogenic. In addition, *MYO7A* c.462C>A (p.C154X) was found in three normal hearing family members, including the father and two grand-daughters (I:1, III:1, and III:3), while *MYO7A* c.73G>A (p.G25R) was found only in the mother (I:2) (Figure 1). The novel c.462C>A mutation in *MYO7A* was absent from 219 unrelated Chinese controls. Both amino acids are highly conserved across species (Figure 4B). The mutation of 73G>A causes a glycine to arginine change, and the 462C>A creates a premature stop codon at the position of amino acid 154 of the large myosin VIIa protein of 2215 amino acids.

SIFT and Polyphen2 were used as a filter to predict how the identified amino acid substitutions would affect protein function considering sequence homology and the physical properties of amino acids. Both *MYO7A* c.73G>A (p.G25R) and c.462C>A (p.C154X) were predicted to be damaging (according to GenBank accession number NM_000260.3) [9], [30].

Mutation c.462C>A is a novel mutation (http://www.umd.be/MYO7A/) located within exon 4 that results in the nonsense mutation C154X. The premature stop codon apparently activates the nonsense-mediated mRNA decay response, leading to a decrease in *MYO7A* mRNA expression (Figure 4A). It is predicted that the two compound heterozygous mutations in the same subject caused complete dysfunction of myosin VIIa, leading to the observed phenotype in all three affected family members [9].

### Structure modeling of p. G25R

**Figure 5:**
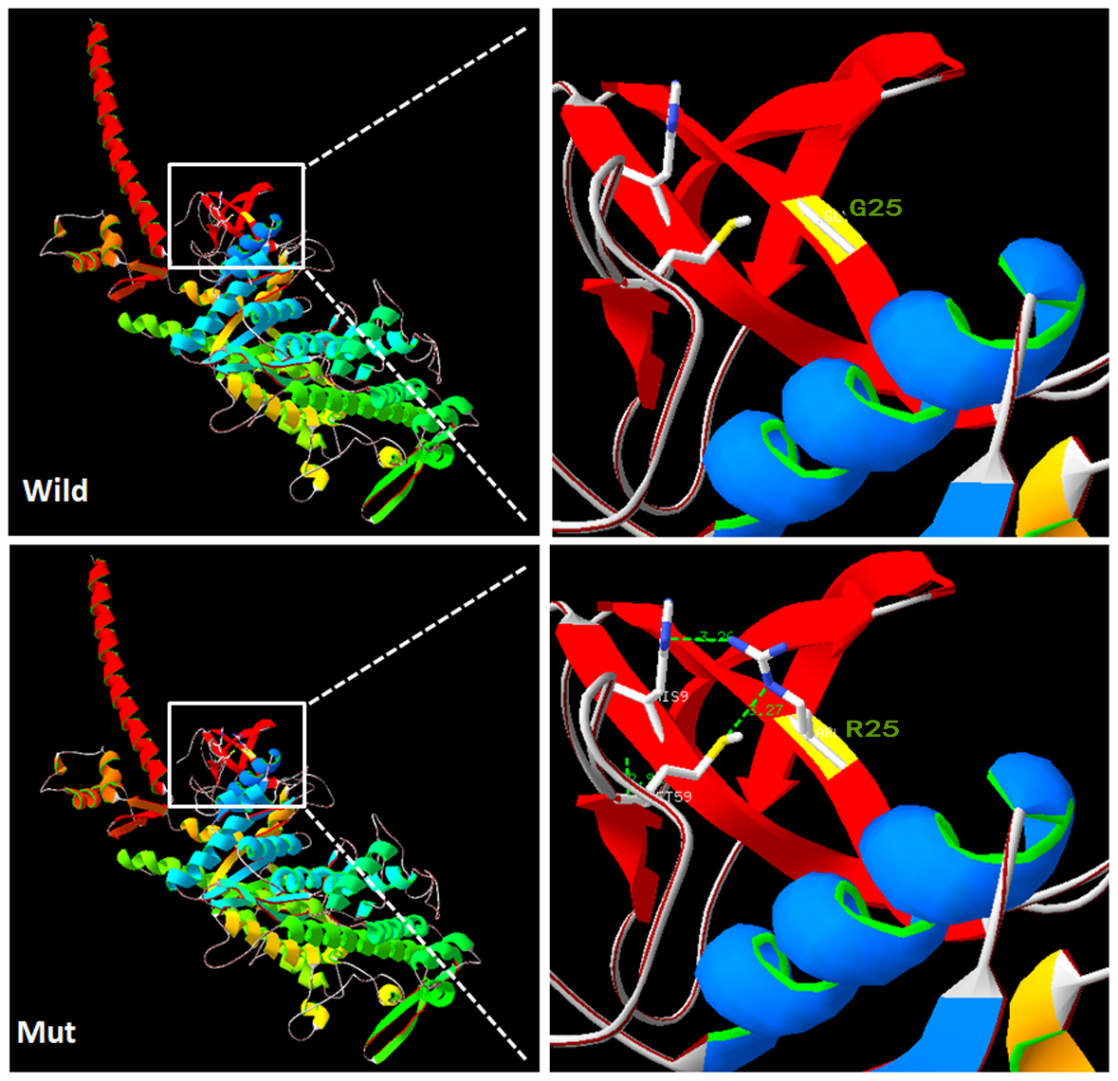
Structure of wild-type and mutant myosin VIIa. A: G25 in the wild-type protein has no side chain to interact with the His9 and Met59. B: The distance (dotted lines) between the long side chain of R25 and the residues H9 and M59 in the mutant protein were less than 3.5Å, which is shorter enough to form new hydrogen bonds (Created by SWISS-MODEL and shown with PY-MOL).

A molecular model of myosin VIIa was constructed based on the crystal structure of myosin V motor (PDB ID: iw7jA). The constructed model covered the target sequence of myosin VIIa (residues 3–769). The sequence identity between the target and template was 39.51%, higher than the average 25%. Using Swiss-PdbViewer 4.1, the mutation was predicted to perturb an amino acid side chain due to the substitution of glycine by arginine and two extra hydrogen bonds. This region of the protein is predicted to be highly hydrophobic, as previously shown in a hydrophobicity plot (Figure 5).

These data, together with the clinical presentation of the four affected siblings and the consistent Mandelian inheritance of the variants in the affected and unaffected members, indicate that the *MYO7A* compound mutations c.73G>A (p.G25R) and c.462C>A (p.C154X) are the cause of USH1 in this family.

## Discussion

Hundreds of different mutations of USH1 are listed in the Universal Mutation Database (UMD) USHbases, a comprehensive set of databases that records pathogenic mutations and unclassified variants in five genes causing USH1 [31]. However, due to the genetic heterogeneity of the disease and the large number of exons of the nine known Usher syndrome genes, the genetic causes for a large proportion of Usher syndrome remain unknown. Targeted deafness gene capture combined with NGS is suited to identify the causative mutations of Usher syndrome and hereditary hearing loss owing to the following advantages: 1) comprehensive coverage of large numbers of genes and large genes associated with the disease; 2) significant cost saving; 3) higher sequencing accuracy because of deeper achievable coverage; 4) a significantly shorter turnaround time and 5) more convincing dataset by excluding other deafness genes.

By sequencing the 366 coding exons and flanking regions of the nine known Usher syndrome genes, Bonnet *et al*. recently found mutations in 91% of the patients tested, improving the molecular diagnosis of Usher syndrome greatly [11]. We speculate that targeted deafness gene capture and NGS provides opportunities to identify causative mutations and new Usher syndrome genes efficiently.

In family 7162, four patients have deafness and vestibular dysfunction and two of them have RP, all symptoms compatible with USH1. Targeted NGS revealed two compound *MYO7A* mutations, c.73G>A and c.462C>A, segregating with disease in this family. The *MYO7A* compound mutations c.73G>A (p.G25R) and c.462C>A (p.C154X) were identified as pathogenic mutations in family 7162 with USH1. Mutation c.73G>A(p.G25R) was reported previously in a Caucasian population and is considered a recessive pathogenic mutation [9]. It located within exon 2 and changes the conserved uncharged hydrophilic Glycine to positively charged hydrophilic arginine at the highly conserved codon 25 in the N-terminal hydrophobic region, upstream from the motor head of myosin VIIa. In addition, the amino acid glycine does not have a side chain and is often found close to or at the surface in loop regions, conferring high flexibility to these regions. Glycine residues are often highly conserved in protein families since they are essential for preserving a particular protein three-dimensional fold. With the p.G25R mutation, a small uncharged residue without a side chain is replaced by a big, hydrophilic, positively charged amino acid. *In silico* analysis indicated a pathogenic effect of this mutation, given that the region where it is located is highly conserved and structure modeling of G25R results in gaining two extra hydrogen bonds (Figure 5). Therefore, the mutation will lead to extra ionic interactions and other possible interactions of the arginine residue in the mutated-type *MYO7A*, such as creating additional hydrogen bonds, loss of structural flexibility conferred by Glycine, or alternation in protein localization.

The stop codon in exon 4 (c.462C>A [C154X]) identified in this study is close to the reported mutation c.448C>T (R150X) [9]. The mutation would lead to a truncated protein lacking 2061 amino acid residues that contain almost all of the important functional domains (Figure 4A). Therefore, the mutant myosin VIIa protein resulted from either mutation might lose the ability to link the proteins in the cell membrane to the proteins in the cytoskeleton, be misfolded, nonfunctional, or be much reduced.

In summary, we report the clinical and genetic characteristics of a non-consanguineous Chinese family (no. 7162) with autosomal recessive USH1. We identified two *MYO7A* compound heterozygous mutations, c.73G>A and c.462C>A, as disease-causing mutations through multiple deafness gene capture, NGS, and bioinformatic analysis. In the future, we will gather more samples from Chinese USH1 patients and determine the molecular background of USH1 in China to provide the patients and their families with precise, early molecular diagnoses, accurate genetic counseling, and optimal rehabilitation.

The English in this document has been checked by at least two professional editors, both native speakers of English. For a certificate, please see: http://www.textcheck.com/certificate/I3NwIG.

## Supporting Information

Table S1 **List of 131 Deafness genes.**
(DOCX)
