# Correction: Novel Compound Heterozygous Mutations in *MYO7A* Associated with Usher Syndrome 1 in a Chinese Family

**DOI:** 10.1371/journal.pone.0137883

**Published:** 2015-10-01

**Authors:** Xue Gao, Guo-Jian Wang, Yong-Yi Yuan, Feng Xin, Ming-Yu Han, Jing-Qiao Lu, Hui Zhao, Fei Yu, Jin-Cao Xu, Mei-Guang Zhang, Jiang Dong, Xi Lin, Pu Dai

The following information is missing from the Competing Interests section: “Xi Lin owns stocks in Otogenetics Corporation, which may be a source of potential conflict of interest for materials presented in this paper. This does not alter our adherence to *PLOS ONE* policies on sharing data and materials.”
