# Supplementary material for: Novel Compound Heterozygous Mutations in MYO7A Associated with Usher Syndrome 1 in a Chinese Family
Source: PLoS One. 2014 Jul 31;9(7):e103415. doi: 10.1371/journal.pone.0103415 (PMC4117490; doi:10.1371/journal.pone.0103415)
Supplement: Table S1 — List of 131 Deafness genes. (DOCX) [file pone.0103415.s001.docx]

Table S1 131 deafness genes

| ACTB | FGF3 | MYO15A | SPINK5 | MTTS2 | ILDR1 |
| --- | --- | --- | --- | --- | --- |
| ACTG1 | GATA3 | MYO1A | STRC | MTTL2 | MSRB3 |
| ATP6V1B1 | GJA1 | MYO1C | TBL1X | FGFR3 | NDP |
| BCS1L | Gjb1 | MYO1F | TCF21 | mir96 | SNAI2 |
| BSND | GJB2 | MYO3A | TECTA | mir182 | SERPINB6 |
| CATSPER2 | GJB3 | MYO6 | TFCP2 | mir183 | SIX1 |
| CCDC50 | GJB4 | MYO7A | TIMM8A | Gjb2 10.6K | SIX5 |
| CDH23 | GJB6 | NR2F1 | TMC1 | GRXCR1 | TPRN |
| CLDN14 | GRHL2 | OTOA | TMIE | USH2C | FAS |
| COCH | GSTP1 | OTOF | TMPRSS3 | USH2A | EDNRA |
| COL11A2 | JAG1 | OTOR | TMPRSS5 | USH1G | EDNRB |
| COL9A3 | KCNE1 | PAX3 | TRIOBP | USH3A | MITF |
| CRYM | KCNJ10 | PCDH15 | USH1C-a-b | HGF | ECE1 |
| DFNA5 | KCNQ4 | PDZD7 | WFS1 | TJP2 | FKH10 |
| DFNB31 | KIAA1199 | PMP22 | MTTL1 | FOXI1 | HAL |
| DFNB59 | LHFPL5 | POU3F4 | MTTI | ATP6V1B2 | PTPRQ |
| DIAPH1 | LHX3 | POU4F3 | MTTQ | PRPS1 |  |
| DSPP | LRTOMT | RDX | MTTM | SMPX |  |
| ERCC2 | MARVELD2 | SLC17A8 | P2RX2 | COL9A2 |  |
| ERCC3 | MTAP | SLC26A4 | MTTS1 | KCNQ1 |  |
| ESPN | LOXHD1 | SLC26A5 | MTTD | CEACAM16 |  |
| ESRRB | MYH14 | SLC4A11 | MTTK | GIPC3 |  |
| EYA4 | MYH9 | SOX2 | MTTH | GPSM2 |  |
